# P-2061. Beyond Distance: Social Barriers Influence Long-acting Antiretrovirals Location Preference Among People with HIV in Rural Areas

**DOI:** 10.1093/ofid/ofaf695.2225

**Published:** 2026-01-11

**Authors:** Emmanuel Nazaire Essam Nkodo, Renae Furl, Elizabeth Lyden, Dan Cramer, Jennifer O’Neill, Maureen Kubat, Heather Saarela, Titilola Labisi, Nada Fadul

**Affiliations:** University of Nebraska Medical Center, Omaha, NE; University of Nebraska Medical Center, Omaha, NE; University of Nebraska Medical Center, Omaha, NE; University of Nebraska - Medical Center, Omaha, Nebraska; University of Nebraska Medical Center, Omaha, NE; University of Nebraska Medical Center, Omaha, NE; University of Nebraska - Medical Center, Omaha, Nebraska; Kaiser Permanente, Los Angeles, California; University of Nebraska Medical Center, Omaha, NE

## Abstract

**Background:**

People with HIV (PWH) living in rural and remote areas are disproportionately impacted by social and structural barriers such as stigma, poverty, and transportation issues. While long-acting injectable antiretrovirals (LAI ART) have the potential to improve adherence, they can exacerbate barriers by increasing travel burden to clinics (monthly or every 2 months). Our study aimed to investigate location preference for LAI ART in PWH living remotely from the clinic and factors associated with preference.

**Figure:**
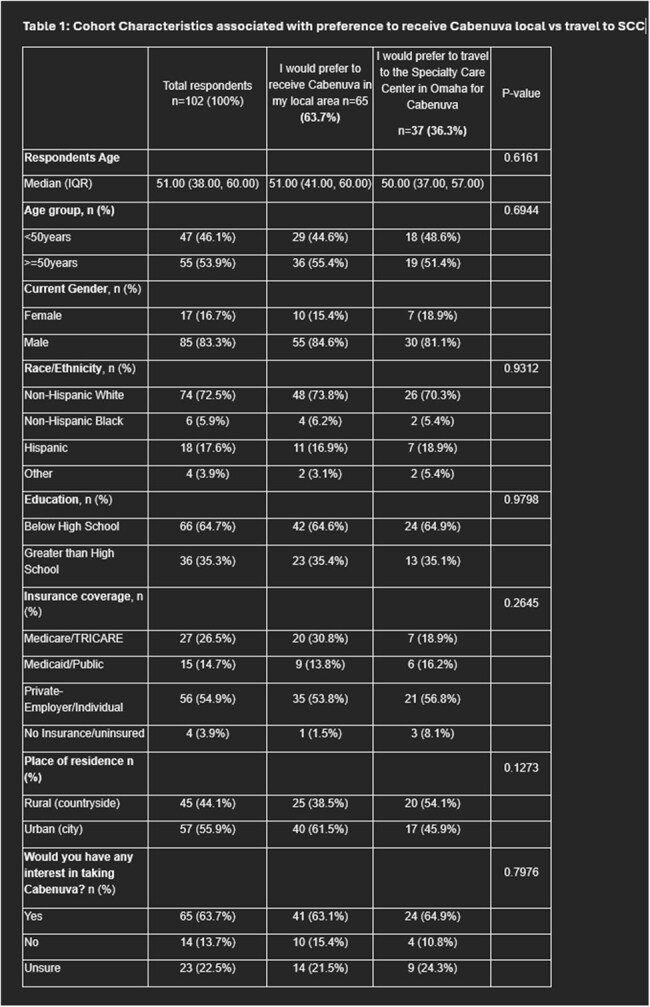
Cohort Characteristics associated with preference to receive Cabenuva local vs travel to SCC

**Figure:**
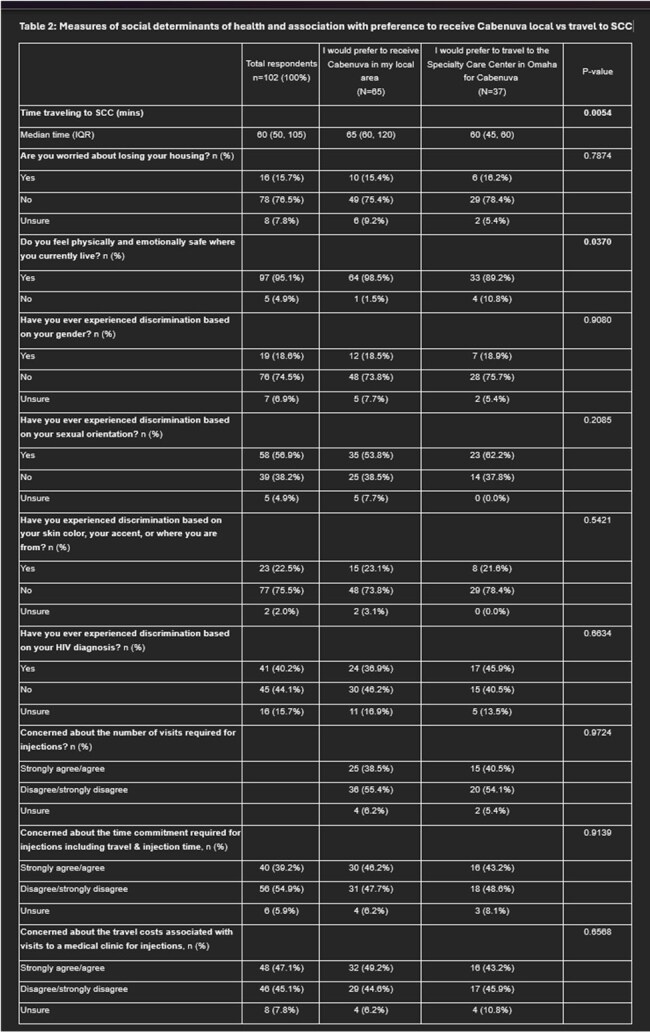
Measures of social determinants of health and association with preference to receive Cabenuva local vs travel to SCC

**Methods:**

We conducted an online cross-sectional survey of PWH established on oral ART but naïve to LAI-ART who live outside the Omaha metro area and travel to the University of Nebraska Medical Center’s Specialty Care Center (SCC) for care. We assessed interest in and preference to receive LAI-ART locally vs. at SCC and the association with social determinants of health (SDOH), travel time, and stigma.

**Results:**

102 PWH completed the survey (Table 1), with 44% describing their residence as rural, and 39% preferring to receive LAI-ART locally vs. 54% at SCC (p=0.127).  Among those who preferred the SCC (37), 30% (11) refused to receive LAI-ART locally, and 22% (8) were unsure. Among these two subgroups (19 respondents), 8 reported “privacy concerns”, mentioning “small town gossip” as the reason for not receiving injections locally. Overall, measures of SDOH showed that feeling physically and emotionally safe where they live (p=0.03) and time to travel to the SCC (IQR 60-120 min) (p=0.00) were associated with preference to receive LAI ART locally (Table 2). Experience of discrimination was common, specifically based on HIV diagnosis (40.2%) and sexual orientation (55.4%), the latter being associated with interest in LAI-ART (p =0.031).

**Conclusion:**

Beyond the obvious time/distance issues associated with the frequent clinic visits to receive injections, our findings underscore the role of SDOH in interest in and location preference for LAI-ART. Tailored implementation strategies in rural settings are crucial to ensure LAI-ART adoption does not inadvertently amplify existing social and structural disparities.

**Disclosures:**

Dan Cramer, APRN-NP, Viiv Healthcare: Grant/Research Support Heather Saarela, BSPH, Viiv Healthcare: Grant/Research Support Nada Fadul, MD, ViiV Healthcare: Advisor/Consultant|ViiV Healthcare: Grant/Research Support

